# Longitudinal evaluation of RTVue optical coherence tomography in patients with glaucoma and suspected glaucoma and stable visual fields

**DOI:** 10.5935/0004-2749.2021-0407

**Published:** 2023-03-08

**Authors:** Valdenir Ribeiro Júnior, Marcos P. Ávila, Cristiane F. Ribeiro, Leopoldo Magacho

**Affiliations:** 1 Ophthalmology Department, Universidade Federal de Goiás, Goiânia, GO, Brazil; 2 VER Hospital de Olhos, Goiânia, GO, Brazil

**Keywords:** Imaging diagnosis/methods, Optical disk/pathology, Nervous fibers/pathology, Glaucoma/diagnosis, Tomography, optical coherence, Diagnóstico por imagem/métodos, Disco óptico/patologia, Fibras nervosas/patologia, Glaucoma/diagnóstico, Tomografia de coerência óptica

## Abstract

**Purpose:**

To longitudinally compare isolated structural parameters obtained using RTVue optical coherence tomography in patients with glaucoma and suspected glaucoma with stable visual fields.

**Methods:**

All patients were required to have a reliable SITA Standard 24-2 Humphrey Visual Field test. Visual field stability was defined as having <5 points with p<5% and/or having no points with p<1% and/or p<0.05% in the glaucoma progression analysis comparison graph. Furthermore, the glaucoma assessment strategy was used in optical coherence tomography.

**Results:**

The study included 75 eyes from 75 patients, 43 of which had glaucoma and 32 had suspected glaucoma. The mean visual field intervals were 29.57 ± 9.65 months between the first and third tests. No visual field parameter variations (mean deviation, pattern standard deviation, and visual field index) and no retinal nerve fiber layer or optic disk parameter variations between the first and third tests were observed (p>0.05 for all), and no retinal nerve fiber layer parameter variations throughout the study were observed, except for optic disk parameters presenting with cup volume changes (p=0.004). However, ganglion complex cells presented a progressively decreased average ganglion cell complex parameter, with a variability of -0.98% ± 3.71% (p=0.04) between the first and third tests. By contrast, the global loss volume progressively increased throughout the study, with a variability of 14.71% ± 44.52% (p=0.04) between the first and third tests. The inferior ganglion cell complex parameter was significantly decreased between the first and third tests (p=0.02).

**Conclusion:**

The present findings suggest that patients with glaucoma or suspected glaucoma with stable visual fields may present structural ganglion complex cell progression as assessed using RTVue optical coherence tomography.

## Abstract (pt)

**Objetivo:**

Comparar longitudinalmente os parâmetros estruturais isolados obtidos através da tomografia de coerência óptica RTVue em pacientes glaucomatosos e suspeitos de glaucoma com campos visuais estáveis.

**Métodos:**

Todos os incluídos deveriam ter Campimetria Computadorizada Humphrey Sita Standard 24-2 confiáveis. A estabilidade campimétrica foi definida se apresentassem menos de cinco pontos com p<5% e/ou nenhum ponto com p<1% e/ou p<0,05% no gráfico de comparação do *Glaucoma Progression Analysis*. Para a tomografia de coerência óptica, foi utilizado a estratégia de avaliação para glaucoma.

**Resultados:**

Foram incluídos 75 olhos de 75 pacientes: 43 com glaucoma e 32 suspeitos. A média dos intervalos do campo visual entre o 1^o^ e 3^o^ exame, foi de 29,57 ± 9,65 meses. Não houve variação para os parâmetros do campo visual (desvio médio, desvio padrão e índice da função visual) entre o primeiro e o último exame (p>0,05 para todos). Não houve variação dos parâmetros da camada de fibras nervosas da retina ao longo do estudo, enquanto que para os parâmetros do disco óptico, apenas *cup volume* apresentou mudança (p=0,004). Em relação à camada de células ganglionares da retina, notou-se uma redução progressiva na espessura média da *Ganglionar Complex Cells* com uma variabilidade entre o primeiro e último exame de -0,98 ± 3,71% (p=0,04). Quanto ao *Global loss volume*, houve um aumento progressivo ao longo do estudo com uma variabilidade entre o primeiro e último exame de 14,71 ± 44,52% (p=0,04). O parâmetro inferior do *Ganglionar Complex Cells* também reduziu significativamente entre o 1^o^ e 3^o^ exames (p=0,02). Os demais parâmetros da tomografia de coerência óptica RTVue se mantiveram estáveis entre o 1^o^ e 3^o^ exames.

**Conclusão:**

Os presentes achados sugerem que pacientes glaucomatosos ou com suspeita de glaucoma e com campos visuais estáveis, podem apresentar progressão estrutural na camada de células ganglionares da retina avaliada por meio da tomografia de coerência óptica RTVue.

## INTRODUCTION

Despite studies of structural and functional defects in glaucoma using several examination techniques, the correlation between them has led to conflicting results in some cases^(1^,^2)^. Specifically glaucoma progression can be seen as either an increased functional loss in a series of visual fields (VFs) or as an increased structural loss in the optic nerve head or retinal nerve fiber layer (RNFL). The concept of “ganglion cell dysfunction,” rather than retinal ganglion cell (RGC) death, may partly explain these findings, wherein perimetric damage was confirmed by decreased VF sensitivity in those with an optic disk (OD) without evident structural changes^(3)^. Thus, within a more current concept, the detection of structural and functional changes can occur simultaneously, or at diffe-rent times^(4)^.

Despite previous findings, the relationship between structural and visual function losses at each stage of glaucoma and/or suspected glaucoma remains unclear. Furthermore, current methods that can assess function cannot detect changes in early progression. Thus, the combination of structural and functional assessment results in a greater number of diagnostic variables in glaucoma or suspected glaucoma assessment, ideally resulting to an increase in the accuracy of glaucoma diagnosis and progression follow-up^(5^,^6)^.

Despite studies on the longitudinal relationship between functional VF and structural OCT measurements^(7^,^8)^, no studies have reported the behavior of structural parameters against stable functional damage in patients with glaucoma and suspected glaucoma. Thus, this study was conducted to address this concern.

## METHODS

This study included all patients undergoing glaucoma evaluation by the same glaucoma specialist at VER Eye Hospital; for 5 years, they were consecutively and retrospectively chosen from the date of protocol approval by the research ethics committee (REC). This study began after obtaining approval from the REC of the Federal University of Goiás, GO, Brazil, and from the VER Eye Hospital Ethics Committee.

All patients were at least 18 years old and had at least three reliable SITA Standard 24-2 Humphrey VF tests (Humphrey/Zeiss, San Leandro, CA, USA), with RTVue OCT (Optovue, Fremont, CA, USA) performed at least 12 months apart. VF examinations should have lasted for <7 min^(9)^, with false-positive, false-negative, and fixation loss rates of <20%^(10)^. The first VF test was excluded from the analysis to reduce the VF learning effect. If both eyes of the same patient were eligible for the study, only the right eye was included.

Then, VF stability tests were independently analyzed by two masked glaucoma specialists. The tests were considered stable if they had <5 points with p<5% and/or no points with p<1% or p<0.05% in the (GPA) in any test performed during the follow-up period^(10)^. Meanwhile, if for any reason a patient had no GPA, the clinical judgment of both specialists was used to define whether they had stable VF tests. If agreement was not reached between examiners, the patient was excluded from the study.

RTVue OCT was obtained from patients’ charts in three maps: for the RNFL, optic nerve head, and ganglion cell complex (GCC) parameters. OCT image quality assessment was based on the quality score provided by the device itself (signal strength [SSD]), which should be >45, and all tests were performed by a trained and experienced technician. In addition, VF and OCT exa-minations of each patient at each visit should be performed 60 days apart at most to be included in the study.

The inclusion criteria for patients with glaucoma were as follows: glaucomatous OD with cup-disk (C/D) ratio ≥0.7 with a nonhomogeneous neural rim, C/D ratio >0.5 with localized or complete nervous tissue absence, with or without VF defect according to the Hodapp-Parrish-Anderson criteria^(11)^, regardless of the intraocular pressure (IOP) levels. Meanwhile, the inclusion criteria for patients with suspected glaucoma were as follows: increased cupping without characteristic signs of glaucoma (described above), with normal VF and IOP ≤21 mmHg in at least three measurements taken on different days. Patients with ocular hypertension (IOP >21 mmHg without functional or structural glaucomatous damage) were also considered to have “suspected glaucoma”.

On the contrary, the exclusion criteria for both groups were any conditions that could interfere with the results of the studied tests, including high ametropia, amblyopia, fundus disorders such as macular scars, cataracts, unreliable VF tests, low quality tests (OCT with SSD <45), secondary glaucoma, previous history of intraocular surgery, or any eye surgery (including for glaucoma) performed during the follow-up period.

Data obtained were processed using the IBM SPSS Statistics for Windows, version 21.0 (IBM Corp., Armonk, NY, USA). The Kolmogorov-Smirnov test was used to verify the normality of the quantitative variables, in which variables with p-values >0.05 were considered to have normal distribution. Moreover, VF and OCT parameter variations were analyzed as quantitative measurements and presented as percentages. Then, the tests were compared in groups and pairs relative to the first test, verifying where possible statistically significant variations could occur.

Quantitative variables were presented as means, standard deviations, medians, and minimum and maximum values. The Wilcoxon test was used to compare the means of quantitative variables between two moments, whereas the Friedman test was used for four moments. On the contrary, the Mann-Whitney test was used to compare the distributions of nonparametric quantitative variables between two groups, whereas the Kruskal-Wallis test was conducted for three groups. Furthermore, Spearman’s correlation coefficient was used to verify the correlation between two quantitative variables. All cases for these analyses considered at a 5% significance level (p<0.05).

## RESULTS

The study included 75 eyes from 75 patients, with a mean age of 48.5 ± 15.8 (range, 19-85 years) years. Of these patients,38 were men and 37 were women, 43 of which had glaucoma and 32 had suspected glaucoma, totaling 49 right and 26 left eyes. In addition to their first test, which was considered for baseline, all 75 patients had two VF and OCT tests, of which 67 had three tests. All patients had GPA. The mean interval between the first and second VF tests was 17.4 ± 8.1 months and that between the first and third tests was 29.5 ± 9.6 months. Regarding OCT, the mean interval between the first and second tests was 17.7 ± 8.5 months and that between the first and third tests was 30.8 ± 10.1 months.

Table 1 shows the mean values of each VF parameter and their comparisons in groups and pairs. Considering only the first and last VF tests of all patients, a VFI variation of 1.26% ± 6.6% (p=0.7), more initial damage (MD) variation of -21.9% ± 169.7% (p=0.09), and PSD variation of 18.0% ± 53.4% (p=0.1) were observed.

**Table 1:** Comparison of the mean visual field variables between the three tests

	Test #1	Test #2	Test #3	p-value^*^	p-value^**^ Test #1 vs. #2	p-value^**^ Test #1 vs. #3
VFI (%)	89.52 ± 19.04	91.75 ± 16.29	89.16 ± 19.14	0.02	0.01	0.7
MD (dB)	-4.45 ± 6.51	-3.43 ± 5.52	-4.24 ± 6.43	0.01	0.002	0.09
PSD (dB)	3.66 ± 3.31	3.48 ± 3.41	4.09 ± 3.60	0.4	0.4	0.1

* Friedman test

** Wilcoxon test.

Table 2 illustrates the mean values of each OCT variable in each of the three tests and their comparisons in groups and pairs. The comparison between the first and last tests showed a statistically significant difference in some parameters, with a GCC variation of 1.0% ± 3.7% (p=0.04), Inf. GCC of -1.4% ± 4.5% (p=0.02), and a global loss volume (GLV) of 14.7% ± 44.5% (p=0.04). The other RTVue parameters remained stable (p>0.05) throughout the study.

**Table 2:** Comparison of the values obtained in the three tests with RTVue OCT variables

	Test #1	Test #2	Test #3	p-value^*^	p-value^**^ Test #1 vs. #2	p-value^**^ Test #1 vs. #3
Avg. RNFL^#^	96.22 ± 17.50	98.00 ± 19.50	95.55 ± 17.71	0.08	0.8	0.5
Sup. Avg. ^#^	95.11 ± 17.33	95.84 ± 19.66	93.86 ± 16.70	0.4	0.1	0.2
Inf. Avg. ^#^	96.79 ± 23.46	100.25 ± 21.31	97.23 ± 20.07	0.2	0.2	0.9
Rim volume^##^	0.07 ± 0.14	0.07 ± 0.12	0.05 ± 0.04	0.08	0.06	0.5
Nerve head Vlm^##^	0.10 ± 0.08	0.13 ± 0.16	0.10 ± 0.07	0.3	0.03	0.7
Cup volume^##^	0.50 ± 0.32	0.43 ± 0.27	0.50 ± 0.30	0.004	0.006	0.6
Optic disk area^##^	2.15 ± 0.40	2.13 ± 0.37	2.15 ± 0.39	0.2	0.8	0.3
C/D area ratio	0.68 ± 0.16	0.65 ± 0.19	0.67 ± 0.16	0.08	0.02	0.8
Hor. C/D ratio	0.90 ± 0.09	0.88 ± 0.13	0.89 ± 0.11	0.2	0.1	0.4
Vertical C/D ratio	0.83 ± 0.10	0.82 ± 0.11	0.83 ± 0.11	0.2	0.05	0.3
Rim area^##^	0.66 ± 0.33	0.73 ± 0.42	0.67 ± 0.35	0.1	0.03	0.9
Cup area^##^	1.49 ± 0.46	1.40 ± 0.49	1.48 ± 0.48	0.1	0.04	0.8
Avg. GCC^#^	85.90 ± 11.54	85.66 ± 11.43	85.34 ± 11.60	0.04	0.08	0.04
Sup. GCC^#^	86.00 ± 10.79	86.04 ± 10.40	85.80 ± 10.51	0.3	0.4	0.1
Inf. GCC^#^	85.80 ± 12.75	85.29 ± 13.14	84.89 ± 13.34	0.3	0.08	0.02
FLV (%)	3.44 ± 4.10	3.57 ± 3.94	3.47 ± 4.24	0.3	0.3	0.1
GLV (%)	11.55 ± 10.33	11.85 ± 10.11	12.00 ± 10.32	0.03	0.04	0.04

* Friedman test

** Wilcoxon test

# µm

## mm^2^.

## DISCUSSION

Despite previous reports on the longitudinal relationship between VF functional and OCT structural measurements^(5^,^8)^, the novelty of the present study lies in its reports of the behavior of structural parameters in the case of stable functional damage in these patients.

VF and OCT were reported to have different progression speeds^(12)^ in patients with glaucoma. Structural damage tends to be linear, whereas functional damage measured by VF does not^(6)^. Instead, it usually presents as a slowly measured lesion that then intensifies, suggesting that patients with an initial stable VF may theoretically be progressing at a rate slower than its ability to detect significant changes, thus remaining unnoticed.

On average, the study patients had initial glaucoma^(13)^, presenting with an MD of -4.45 ± 6.51 dB in the first test and -4.24 ± 6.43 dB (p=0.09) in the last test. Patients with initial VF damage were chosen because functional lesions are relatively insensitive to change detection in the early stages of the disease^(14)^, and by analogy, this is also true for progression detection. If the lack of VF sensitivity for detecting progression at disease onset is likely related to the logarithmic scale used for VF sensitivity measurements, VF could not estimate small amounts of RGC losses in the early stages of glaucoma^(7^,^15)^.

The relationship between structural and functional findings in glaucoma has been previously described^(5^,^6)^. In a more current concept, structural and functional changes can be detected simultaneously or at different times^(4)^, which means that a patient with glaucoma may have structural or functional damage first, or even at the same time.

In this study, the mean VF interval between the first and second tests was 17.48 ± 8.18 months and that between the first and third tests was 29.57 ± 9.65 months, which was possibly an adequate amount of time to detect possible progression in at least a portion of the included eyes. VF progression in glaucoma measured by MD deterioration rates can be clinically significant even when the follow-up period was shorter, i.e., between 12 and 18 months^(16)^.

This study used GPA to determine the presence of VF stability. In some studies, GPA has shown a greater and even earlier sensitivity in glaucomatous progression detection than VFI analysis, which requires a greater number of VF tests. Moreover, GPA aims to remove the examiner’s subjectivity in serial VF evaluation of the VF using an objective method to determine VF stability. The first VF test was used to establish the GPA baseline, including the next two VFs for progression analysis. This definition aimed at relativizing the learning effect expected in VF examinations. However, patients who could not undergo GPA for any reason were subjectively assessed by two masked glaucoma specialists to reduce selection bias.

The VFI comparison considering all VF examinations showed a statistically significant difference (p=0.02), which was not observed in the paired evaluation between the first and third (last) VF tests (p=0.7). This statistical change appeared to be caused by an improvement in the results of the second test, as the VFI results between the first and the last VF tests progressed with a change of 1.26% ± 6.57% (p=0.006). Despite the statistical significance, this minor difference may not have any clinical implication. Some explanations can be postulated, such as long-term fluctuation or even the natural variability in VF examinations, even if performed by healthy, trained, and reliable evaluators^(17)^. On the contrary, this variation was an improvement in absolute VFI values and therefore not a possible progression.

Similar results were found in MD, showing values of -4.45 ± 6.51, -3.43 ± 5.52, and -4.24 ± 6.43 dB in the first, second, and third tests (p=0.02), respectively, and the results between the first and third tests were comparable (p=0.09). For PSD, despite an improved value in the second test (3.48 ± 3.41 dB) compared with the first one (3.66 ± 3.31 dB), further deterioration was noticed in the third test (4.09 ± 3.60 dB); however, no statistical significance was noted (p=0.4).

Structural measurements of OD parameters, including rim volume (p=0.08), nerve head volume (p=0.3), OD area (p=0.2), C/D area ratio (p=0.08), horizontal C/D ratio (p=0.2), vertical C/D ratio (p=0.2), rim area (p=0.1), and cup area (p=0.1), as measured by RTVue OCT, showed no statistically significant changes throughout the study, except for the cup volume (p=0.004). Specifically, cup volume evaluation showed decreased results between the first and second tests (p=0.006); however, no statistical significance was noted in the changed cup volume between the first and last tests^(18^,^19)^.

In addition, OD parameters measured by OCT require the establishment of anatomical OD and cupping boundaries. The cupping boundary is identified as a fixed distance above the line connecting the edges of Bruch’s membrane (BM). RTVue OCT segmentation may underestimate disk dimensions, apparently as a result of assuming some amount of BM at disk edges. This segmentation effect was consistent with the substantially higher C/D ratio reported by the RTVue OCT device in one study^(20)^, consequently also applying to cup volume measurements, which would leave this parameter with low reliability both in the diagnosis and follow-up of patients with patients confirmed or suspected glaucoma.

Structural measurements obtained from Avg RNFL (p=0.08), Sup Avg (p=0.4), and Inf Avg (p=0.2) showed no statistically significant changes throughout the study. By contrast, in the comparison between the three tests with the RTVue OCT variables regarding RGCL, only Avg GCC (p=0.04) and GLV (p=0.03) presented consistent and statistically significant changes throughout the study.

Regarding Avg GCC, a borderline statistical significance was found between the first and second tests (p=0.08), whereas definitive statistical significance was observed between the first and third tests (p=0.04). This observation could be explained by Tan et al., who showed that by isolating the inner retina, the Avg GCC significantly improved glaucoma diagnosis compared with macular retinal thickness^(21)^. Thus, the Avg GCC results in this study could actually indicate small amounts of macular ganglion cell loss and consequent disease progression, which would characterize the included eyes as having slow progression despite stable VF.

A progressive increase in the GLV OCT was also observed throughout the study, with a measurement values of 11.55% ± 10.33%, 11.85% ± 10.11%, and 12.00% ± 10.32% in the first, second, and third tests (p=0.03), respectively. These differences were significant even when comparing the first and second tests or the first and last tests (p=0.04 for both), resulting in an increased GLV of 14.7% ± 44.5% throughout the study (p=0.04). To investigate the ability of different RTVue OCT parameters in early glaucomatous progression detection, Naghizadeh et al.^(8)^ followed 51 glaucoma eyes and 17 healthy eyes and reported that in the RGC complex, FLV and GLV showed a significantly faster progression rate in the glaucoma group than in the control group (p=0.004 and p=0.001, respectively). Another study showed that GLV performed even better in diagnosing perimetric glaucoma than Avg GCC (p=0.01)^(21)^, concluding that the increased FVL and GLV may indicate glaucoma progression. On the contrary, Inf. GCC showed a constant reduction in the first, second, and third tests, without statistical significance (p=0.3). However, the percentage of variation throughout the study showed a decrease of -1.4% ± 4.5% (p=0.02), possibly indicating variability^(18)^. Thus, even with VF stability, it is reasonable to hypothesize that the study patients may have presented structural progression, which is verified by RTVue OCT, especially in GCC evaluation using GLV.

However, these parameters are sensitive to early and mild macular diseases, and a careful evaluation of the macula is necessary before considering increased FLV and GLV as a sign of glaucomatous progression^(22)^. To avoid this possible and crucial bias, the study protocol defined that any other causes of nonglaucomatous visual loss would be considered an exclusion criterion.

Notably, structural and functional evidence reveals that glaucomatous macular damage occurs even in the early stages of glaucoma^(23^,^24)^. This information is clinically important because the macula includes approximately 30% of all RGC and provides information for 55%-60% of the primary visual cortex^(25)^. Some studies have shown macular damage in patients with early glaucoma when appropriate tools were used for assessment, such as 10-2 VF^(24)^ and OCT RGCL scans^(23^,^26^,^27)^.

Despite all these findings, this study has some limitations. First, only 67 of the 75 eyes that had three OCT tests were analyzed in the last VF test. Second, the study lacked a separate analysis between patients with confirmed and suspected glaucoma. The inclusion of all eyes in a single assessment aimed to maintain a more heterogeneous group within the proposed study criteria, with a MD (-5.92 ± 4.06 dB). Moreover, patients with suspected condition that eventually develop glaucoma theoretically present lower glaucomatous damage, resulting to an even less VF ability to detect any significant progression. This mixed inclusion to the assessment of glaucoma and/or progression is also used in other rele-vant articles^(2^,^28)^. Third, retrospective data were used. Despite this, a prospective analysis was performed, thus representing patients with alleged VF stability in daily clinical practice. Fourth, OCT progression software was not used, and it was not compared with the analysis results because it did not allow the assessment of how they would behave in this glaucoma subgroup. Finally, a linear regression from the MD slopes could be a more representative criterion of possible VF progression. However, further examinations are necessary for the use of this statistical method. The use of specialist examination and GPA have, at least partially, reduced this bias.

In conclusion, the present results showed that early structural glaucoma progression based on the RTVue OCT pattern compared with stable VF can be detected earlier using RGCL parameters compared with any OD and RNFL parameters. Notably, RTVue OCT GLV and Avg GCC showed consistent longitudinal variations throughout the study, whereas Inf. GCC worsened between the first and last tests, suggesting possible structural progression despite stable VF.
